# Ipso Facto or Permanent Membership

**Published:** 1914-04

**Authors:** 


**A Monthly Review of Medicine and Surgery**

EDITOR AND PUBLISHER DR. A. L. BENEDICT, 228 Summer St., corner of Elmwood Ave., Buffalo. (Address for all communications. Please make personal and telephone calls before 1 P. M.)

Third, in age, in the western continent. Independent but supports professional organization. News limited mainly to western N. Y. and Penn.

Subscription $2.00 a year; $3.00 for two years in advance. Changes and errors in address or failure or duplication of delivery, should be reported immediately.

YEARLY VOLUME 69 APRIL, 1914 No. 9

### Ipso Facto or Permanent Membership

It occurs to us that this question is at the root of most of the dissatisfaction and lack of harmony in the A. M. A. and its component branches—not that there is so terribly much of either. A large body of ipso facto members or fellows, or whatever they may be called, who have never expressed much personal interest in a society, who do not attend its meetings and who do not directly pay dues, cannot control that society, except by representatives who can not always satisfy the men who do take personal interest in it, who attend its meetings and pay dues. The representatives are nominally responsible to the former class who, obviously, can not direct them so that the responsibility amounts to nothing. Partly because of the very indirect method of electing representatives of representatives and partly because of the well known fact that a body cannot occupy two places at the same time, the delegates of the A. M. A. cannot, even informally, represent the actual, interested members. The latter, without necessarily objecting either to the delegates personally nor to what they do, naturally feel that they ought to have some control of proceedings, themselves. According to Dr. Lydston's view, the whole delegate method of control is illegal, though this requires further litigation. But if his present verdict is sustained, the A. M. A. is up against the problem of an enormous vote by mail or by proxy, which will give no greater satisfaction than the delegate method.

Discounting a few unusually large registrations mainly of casual attendants, the A. M. A. has a total voting strength at the meeting place of somewhere between two and four thousand, representing about double these figures as a genuine total membership. So far as we understand legal conditions, any voluntary organization in this country, of national scope, is a sort of orphan, unless it is adopted by some particular state, but we are inclined to believe that some equitable way could be found to regulate membership, to allow some degree of popular debate and at least some approach to the principles of a pure or at least directly representative democracy, and that these provisions could be made without placing the whole organization in the light of a law breaker. The largest state in the Union, in population and in numbers of the medical profession, New York followed such a method for many years, not always with the harmony of unanimous satisfaction but with the wholesome inharmony of a minority that could express its opinions and hope to grow to a majority. The mere numbers of the real members of the A. M. A. are not so much greater as to render such a plan impracticable for itself. We doubt whether the support of state and national organizations is any more loyal under the present plan of ipso facto membership which carries no actual privileges. On the other hand, the securing of membership in the state and national organizations by passing through a waiting list period in which the principle test was one depending on actual interest, confers an honor which is permanent and which assures intelligent, active interest.

Every fair minded man will concede that there have been few actual abuses in the management of the national and state organizations. The unrest is due to a feeling of impotency on the part of the individual member, to the very human desire to do something one's self, to the American egoism that one has ideas of value that are worth expressing even if they are voted down. And the dissatisfaction is due partly to the fact that it is very difficult for a physician who is really interested in medicine to secure a voice in the management of his professional organization without sacrificing the opportunity to participate in the scientific features of a meeting. Yet he dislikes to feel that he is being managed by physicians who are not particularly interested in medicine, even if he admits that, on the whole, he is being managed in a very capable way. He gets to talking about medical politicians and a wheel within a wheel, and while these terms, even if literally correct, do not necessarily imply corruption or misuse of power or any other evil, they foment discord.

